# Diversity of *Candida* spp. isolated from microbiota and infectious processes in wild mammals: a systematic review

**DOI:** 10.1007/s11259-026-11410-1

**Published:** 2026-07-20

**Authors:** Bianca de Carvalho Sousa, Clara Cecília Azevedo Santana, Isaac Silva Santos Ramos, Lilian Silva Catenacci, André Luis Souza dos Santos, Maria José dos Santos Soares, Raizza Eveline Escórcio Pinheiro

**Affiliations:** 1 Postgraduate Program in Technologies Applied to Animals of Regional Interest (PPGTAIR), Federal University of Piauí (UFPI), Teresina, Piauí Brazil; 2 Postgraduate Program in Computer Science (PPGCC), Federal University of Piauí (UFPI), Teresina, Piauí Brazil; 3 Department of Veterinary Morphophysiology (DMV/CCA/UFPI), Teresina, Piauí Brazil; 4 Postgraduate Program in Microbiology, Institute of Microbiology Paulo de Góes (IMPG), Federal University of Rio de Janeiro (UFRJ), Rio de Janeiro, Brazil

**Keywords:** Fungi, One health, Wildlife, Yeasts

## Abstract

Yeasts of the genus *Candida* are microorganisms of significant relevance in clinical practice, particularly within intensive care units, where they are classified as high-risk pathogens due to their impact on immunocompromised patients. Despite this, fungi frequently remain neglected in comparison to other infectious agents, and their ecological role, specifically concerning wildlife, remains underexplored. Historically, wild animals have been utilized as a source of subsistence by riverside and rural communities, although such consumption has been increasingly regulated in recent years. The interaction among wild animals, domestic species, and humans facilitates microbial exchange across diverse ecological niches, thereby increasing the risk of the transmission of pathogenic microorganisms and underscoring the principles of the One Health concept. Accordingly, this literature review consolidated studies regarding the isolation of *Candida* spp. from commensal sites and infectious processes in wild mammals. Thirty-four studies were analyzed, encompassing 21 *Candida* species across 74 mammalian species. Although *Candida* spp. are present in the vast majority of healthy individuals, their presence in free-ranging animals may serve as an indicator of ecological alterations and anthropogenic influence within these habitats. Furthermore, under specific circumstances, these commensals can cause infections, particularly in cases of immunological fragility. These data underscore the necessity for further research into the role of wildlife as hotspots or sentinels in the dissemination of these yeasts, contributing to the monitoring and management of emerging diseases, as well as to the health and conservation of wild populations.

**Supplementary Information:**

The online version contains supplementary material available at https://doi.org/10.1007/s11259-026-11410-1.

## Introduction

The relationship between humans and animals has become progressively closer over recent decades, driven by factors such as urban expansion, environmental disruption, wildlife trade, and intensification of animal husbandry. As a consequence of these interactions, previously unknown or poorly monitored infectious agents have emerged as significant public health threats. This phenomenon is exemplified by the recent COVID-19 pandemic (Sharma et al. 2021), a disease that was originally identified in wildlife and subsequently spilled over into human populations. This event underscores the critical importance of integrated surveillance and prevention strategies within a One Health framework, recognizing the interconnectedness of human, animal, and environmental health.

In recent decades, the process of animal domestication has facilitated the exchange of genetic information among microbes across different species. Specifically, the rising popularity of unconventional pets, such as birds (Talazadeh et al. 2022) and reptiles (Nardoni et al. 2008), alongside the use of wild animals for bushmeat consumption and the manufacturing of derivatives, has significantly contributed to this proximity. This contact, often unregulated or illegal, exposes human populations to pathogens circulating among animals that occasionally find favorable conditions to cross the interspecies barrier.

Hunting, exotic animal trafficking, and the commercialization of wildlife meat are common practices in various regions of the world, including Brazil, fostering the emergence of new infectious diseases (Gomes 2010). Furthermore, the continuous destruction of natural habitats (driven by activities such as deforestation, mining, and agricultural expansion) contributes to the emergence of these diseases by promoting ecological imbalances that favor species displacement and, consequently, exposure to novel pathogens. Climate change also plays a critical role in this scenario, altering the distribution patterns of microorganisms and vectors, while directly influencing the interaction dynamics between hosts and infectious agents (Gudipati et al. 2020).

A notable example of this complex interaction is *Candida auris* (*Candidozyma auris*), an emerging multidrug-resistant fungus. Recent hypotheses suggest that this yeast may have originated as a plant saprophyte in warm, saline environments, progressively developing thermotolerance and halotolerance. These attributes likely enabled its initial adaptation to birds, particularly those inhabiting coastal environments, and subsequently, its colonization of humans (Garcia-Bustos et al. 2024). Moreover, *C. auris* has developed multiple mechanisms of antifungal resistance, which has drawn increasing attention due to the associated rise in treatment failure and the limited effectiveness of current therapeutic options. Therefore, this potential evolutionary pathway underscores the need for attention to environmental changes and how they shape fungal adaptation.

In this context, *C. auris* represents only one of the diverse species within the genus *Candida*, which comprises more than 200 representatives (GBIF 2023). Among them, *C. albicans* stands out as the most well-known agent, identified in a wide diversity of hosts both as a component of the microbiota and in infectious processes. Moreover, it exhibits multiple well-characterized virulence factors (Macias-Paz et al. 2023), supporting its classification within the World Health Organization’s (WHO) priority fungal pathogen group and underscoring its significant threat to global public health (WHO 2022). Over the last decades, non-*albicans Candida* species have also been isolated from human infections (Presente et al. 2023; Pyrpasopoulou et al. 2025) and domestic animals (Saavedra et al. 2024), with increasing reports of antifungal resistance, highlighting them as significant emerging agents within the One Health context (Whaley et al. 2017; Tortorano et al. 2021).

Studies on animal microbiota allow for a greater understanding of the ecology and the role that microbes play in different organisms. Examples include the isolation and identification of *Cryptococcus* spp. in birds (Siqueira et al. 2022; Taha et al. 2024) and *Histoplasma capsulatum* in bats (Gugnani and Denning 2023). Additionally, other zoonotic agents such as *Leptospira* spp. (Dietrich et al. 2018; Shinya et al. 2021; Balcázar et al. 2024) and *Toxoplasma* spp. (Aguirre et al. 2019; Almería et al. 2021) have been isolated from wildlife, evidencing the role of this fauna as potential reservoirs for pathogens of importance to One Health.

In this scenario, it is fundamental to consider the "One Health" approach as a framework for the integrated surveillance of the *Candida* genus in mammals. The growing identification of species with zoonotic potential (some multidrug-resistant and capable of adapting to multiple hosts) indicates the need to expand studies on their ecology, transmission routes, and adaptability to new niches. Investigations integrating clinical, environmental, and molecular data may clarify the role of animals (primarily wildlife) in the maintenance and dissemination of these yeasts, contributing to more effective preventive strategies and to the understanding of fungi as key elements in interconnected health systems.

## Methodology

The present review followed the guidelines proposed by the Preferred Reporting Items for Systematic Reviews and Meta-Analyses, PRISMA 2020 Guidelines (Page et al. 2021). A systematic search was performed across PubMed, Scopus, SciELO, Web of Science, and BVS Veterinária. For PubMed specifically, we opted to use MeSH descriptors such as Candida OR Yeasts OR Mycobiome OR Mycoses OR Fungal infection AND Animals, Wild OR Wildlife OR Mammals AND wild OR feral OR sylvatic OR free-ranging. We adapted the terms for the other databases, as detailed in Supplementary Table 1. Additionally, 10 studies were identified in a preliminary search using free Boolean operators: (wild animal) OR (wildlife) AND (*Candida*) and were manually included, as they fulfilled the inclusion criteria but were not captured by the refined strings. A temporal filter was applied, considering only publications from the year 2000 onwards, due to the advancements in identification techniques and recent taxonomic changes involving species previously classified under the genus *Candida* (Kidd et al. 2023; Liu et al. 2026). Nevertheless, all species that historically belonged to this genus were considered, regardless of recent nomenclatural alterations, aiming to maximize the number of included studies, as not all authors have adopted the updated taxonomic classifications. For the purposes of this review, we retained the genus *Candida* for all findings, clarity and consistency with reservations regarding new names where necessary.

Initial screening was conducted by a single reviewer, followed by independent full-text assessment and data extraction by two reviewers to ensure consistency. Inclusion criteria were established to encompass studies that identified and/or isolated *Candida* spp. in wild mammals, whether as pathological agents in infectious processes or as constituents of the commensal microbiota. The following exclusion criteria were established to ensure the specificity of this systematic review: (1) publication types such as book chapters, editorials, review articles, and systematic reviews; (2) studies published in languages other than English, Spanish, or Portuguese; (3) studies focusing exclusively on immunological assays without pathogen isolation; (4) research primarily dedicated to the comparison of diagnostic methods (e.g., biochemical, molecular, or proteomic assays) that did not provide novel primary data regarding fungal occurrence, ecology, or clinical manifestations in wildlife; (5) studies that failed to provide taxonomic identification of the mammalian host at the species level, preventing ecological niche analysis; and (6) reports that did not explicitly describe the isolation or identification of species within the genus *Candida* in wild mammals, even if other yeasts or filamentous fungi were documented.

Citations were exported to the Zotero® reference management software for duplicate removal. Extracted data were organized in a standardized spreadsheet (Google Sheets), encompassing: author names, publication year, study location, *Candida* species identified, host mammalian species, identification and confirmation methods, and the co-isolation of other non-*Candida* microbes. The animals were grouped taxonomically to facilitate scientific interpretation. Graphs were generated using GraphPad Prism.

## Results

**Fig. 1:**
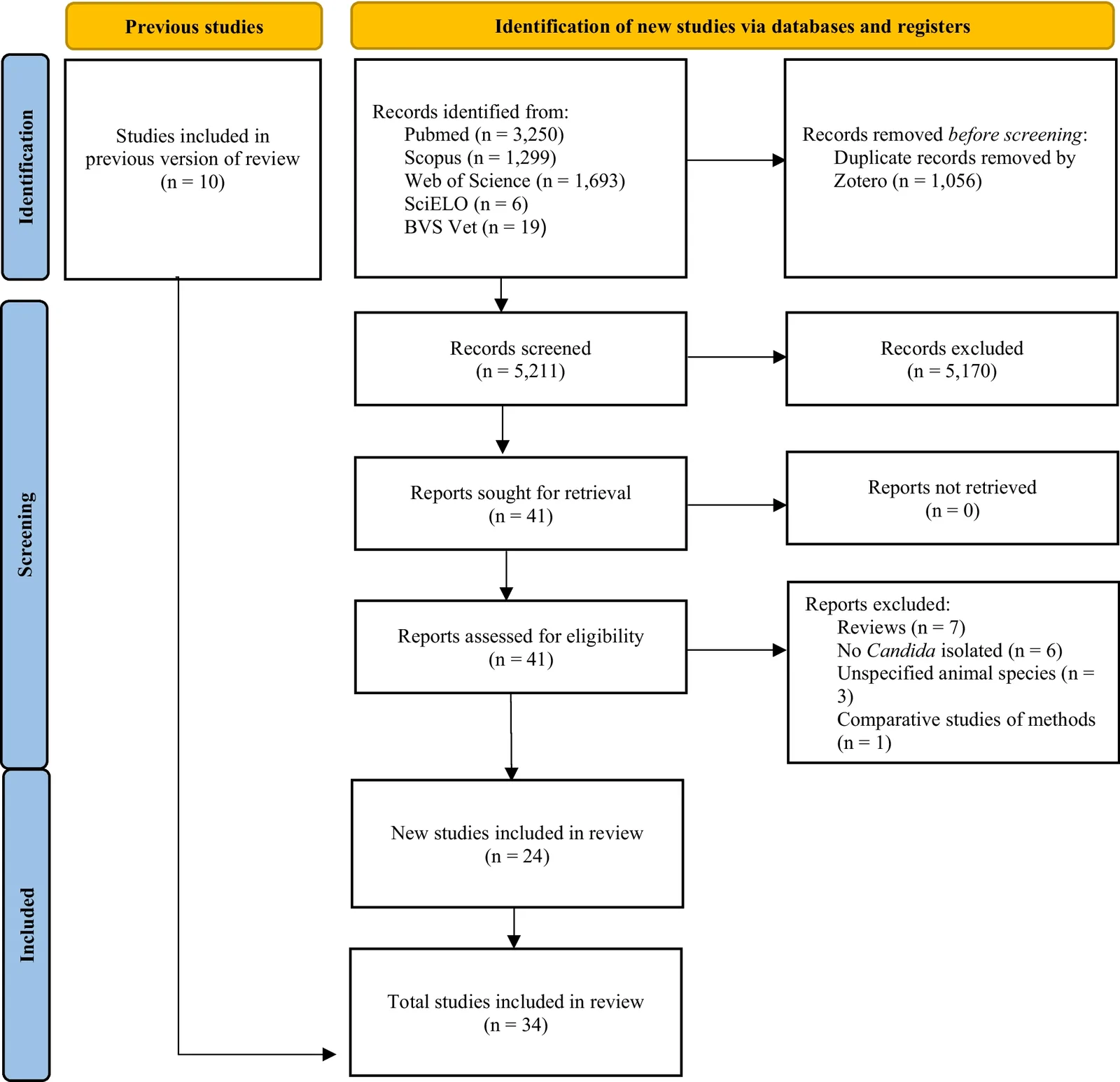
PRISMA flow diagram of the literature search and selection process. A total of 34 studies were selected from 6,267 records after applying the screening and exclusion criteria, with 10 additional studies included from a previous search

The initial search across five electronic databases yielded a total of 6,267 records. After the removal of 1,056 duplicates, 5,211 unique records remained for screening. During the first stage of screening, 5,170 studies were excluded based on title and abstract. Subsequently, 41 full-text articles were sought for retrieval and assessed for eligibility. Following a comprehensive evaluation, 17 reports were excluded based on the exclusion criteria described in the flowchart (Fig. 1), resulting in 24 new studies from the refined search. Additionally, 10 studies were manually included, as they fulfilled all inclusion criteria despite not being captured by the database-specific strings. This culminated in a final selection of 34 studies for this systematic review, which 22 addressed the commensal microbiota of animals (Table 1), while 12 were related to infectious processes (Table 2).

**Table 1:** Summary of studies reporting *Candida* spp. in the commensal microbiota of wild mammals

Animal species	*Candida* species	Anatomical site	Location	Reference
Aquatic mammals				
Bottlenose dolphin (*Tursiops truncatus*)	*Candida albicans; C. tropicalis; C. guilliermondii; C. lusitaniae; C. parapsilosis; C. rugosa; C. famata*; *C. glabrata*^a^*; Candida sp*	Blowhole and anal *swabs* and feces	Florida, North Carolina and Texas, USA	Buck et al. (2006)
Hawaiian monk seal (*Monachus schauinslandi*)	*C. albicans*	Oral and nasal *swabs*	Hawaii, USA	Kissel et al. (2011)
Bottlenose dolphin (*Tursiops truncatus*)	*C. albicans; C. glabrata; C. rugosa; C. tropicalis; C. saitoana*^b^	Blowhole, gastric fluid, and anal *swab*	Southeastern USA	Morris et al. (2011)
Dwarf sperm whale (*Kogia sima*); Amazonian manatee (*Trichechus inunguis*); West Indian manatee (*Trichechus manatus*)	*C. tropicalis*	Oral, nasal, genital, rectal, skin, and blowhole *swabs*	Ceará, Brazil	Cordeiro et al. (2015)
Southern elephant seal (*Mirounga leonina*); Weddell seal (*Leptonychotes weddellii*)	*C. parapsilosis*	Oral and nasal* swabs*	Argentina Antarctica	Brito Devoto et al. (2022)
Amazonian manatee (*Trichechus inunguis*)	*C. orthopsilosis; C. parapsilosis; Candida* spp.	Nasal cavity and rectum	Manaus, Amazonas, Brazil	Colombo et al. (2024)
Artiodactyls				
Mountain gazelle (*Gazella gazella*); Arabian sand gazelle (*Gazella subgutturosa marica*)	*C. albicans*	Swabs from mouth, nose and rectum	Riad, Saudi Arabia	Al-Saggaf et al. (2011)
Wild boar (*Sus scrofa*)	*C. albicans; C. krusei; C. parapsilosis; C. fermentati; C. guilliermondii; C. metapsilosis; C. lusitaniae; C. lambica*; *C. slooffiae*^c^	Fecal sample	South of Italy	Rhimi et al. (2022)
Golden takin (*Budorcas taxicolor*)	*C. albicans*	Feces	China	Ma et al. (2024)
Collared peccary (*Pecari tajacu*)	*C. albicans; C. krusei; C. parapsilosis; C. glabrata; C. tropicalis; Candida* sp.	Oral *swabs*	Amazonas, Brazil	Santos et al. (2026)
Carnivores				
Giant panda (*Ailuropoda melanoleuca*)	*C. tropicalis*	Feces	China	Tun et al. (2014)
Polar bear (*Ursus maritimus*)	*C. sake; C. tropicalis*	Feces	Islands in the Kara and Barents Seas (Arctic), Russia	Vecherskii et al. (2023)
Chiropterans				
Flying fox (*Pteropus giganteus*)	*Candida* spp	Bolus and guano	Lahore, Pakistan	Gulraiz et al. (2017)
Eulipotyphla				
European hedgehog (*Erinaceus europaeus*)	*C. albicans;* C*. guilliermondii*; *C. lipolytica*	Oral and rectal *swabs*	Italy	Brustenga et al. (2025)
Primates				
Western lowland gorilla (*Gorilla gorilla gorilla*)	*Candida* spp.	Eyelid and bulbar conjunctiva	Texas, USA	Liang et al. (2005)
Black lion tamarin (*Leontopithecus chrysopygus*)	*C. guilliermondii; C parapsilosis; C. lusitaniae*; *C. tropicalis, C. famata, C. humicola*; *C. rugosa; C. colliculosa; C. albicans*, *C. magnoliae; C. krusei; Candida* spp.	Nasal, oral, and rectal microbiota	São Paulo and Rio de Janeiro, Brazil	Carvalho et al. (2014)
Cynomolgus macaques (*Macaca fascicularis*)	*Candida* sp	Oral and fecal samples	Thailand	Sawaswong et al. (2020)
Rodents and lagomorphs				
Red-rumped agouti (*Dasyprocta leporina*)	*Candida* spp.	Nasal *swab*	Trinidad and Tobago	Suepaul et al. (2016)
Eastern gray squirrel (*Sciurus carolinensis*)	*C. albicans*	Oral, rectal, and vaginal *swabs*	Umbria, Italy	Cruciani et al. (2022)
39 species divide into 5 rodents families (Heteromyidae, Cricetidae, Muridae, Sciuridae, Geomyidae) and 1 lagomorph (Leporidae)	*Candida* sp.	Lung tissues	Arizona, California and New Mexico, USA	Salazar-Hamm et al. (2022)
Lowland paca (*Cuniculus paca*)	*C. albicans*; *Candida* spp	Hair and skin samples	Acre, Brazil	Oliveira et al. (2023)
North American porcupine (*Erethizon dorsatum*)	*C*. *albicans*; *C. famata*; *Candida spp*	Quill	New York and Connecticut, USA	Sokolik et al. (2024)

^a^In this report, *C. famata* and *C. glabrata* were reported as *Torulopsis famata* and *Torulopsis glabrata*

^b^In this report, *C. saitoana* was reported as *Torulopsis candida*

^c^In this report, *C. lambica* and *C. slooffiae* were reported as *Pichia fermentans* and *Kazachstania slooffiae*

**Table 2:** Studies identifying *Candida* spp. from infectious processes in wild mammals

Animal species	*Candida* species	Description case	Location	Reference
Aquatic mammals				
California sea lion (*Zalophus californianus*); Harbor-seal (*Phoca vitulina*)	*Candida zeylanoides, C. lipolytica*	Fungal dermatitis, with erythematous areas on the fins, snout, periorbital region, and trunk. Other agents were also isolated, such *Malassezia* sp and *Trichophyton mentagrophytes*	Tennessee, USA	Pollock and Ramsay (2000)
Harbor-seal (*Phoca vitulina*)	*C. albicans*	Ulcerative keratitis	Florida, USA	Borkowski et al. (2007)
Southern right whale (*Eubalaena australis*)	*C. zeylanoides*	Animal found dead on the coast; *Candida* was cultured and identified by PCR from skin samples, as well as *Filobasidiella neoformans* var. *neoformans*	Western Cape, South Africa	Mouton et al (2009)
Artiodactyls				
Wild boar (*Sus scrofa*)	*C. albicans*	Yellow-green fungal growth masses on the mucosa and cartilage surface; multifocal subcutaneous abscesses on the maxillary surface. Mycotic rhinitis. Also positive for Porcine circovirus, *Pneumocystis* sp, *Aspergillus fumigatus, A*. *flavus* and a lot of bacteria	Rio Grande do Sul, Brazil	Zlotowski et al. (2011)
Carnivores				
Siberian tiger (*Panthera tigris altaica*)	*Candida spp*	Ulcerative pharyngitis, necrotic tonsillitis, catarrhal to pseudomembranous gastroenteritis, hemorrhagic edema, multifocal necrosis in the liver and pancreas; diagnosed with canine distemper. Also isolated *Clostridium* sp, *Campylobacter coli* and *E. coli*	Zagreb, Croatia	Konjević et al. (2011)
Sumatra tiger (*Panthera tigris sumatrae*)	*C. albicans*	Chronic nephritis and necrotizing pyelonephritis. Also isolated *E. coli* from kidneys as well	Jackson, Mississippi, USA	Wilson et al. (2024)
Eulipotyphla				
European hedgehog (*Erinaceus europaeus*)	*C. albicans*	Severe necrotic glossitis, stomatitis, and death. Hemolytic *E.coli* was isolated too	Great Britain	Barlow et al. (2012)
Primates				
Chimpanzee (*Pan troglodytes*)	*Candida* spp.	Cutaneous lesions, dry skin, intense pruritus (itching), and areas of alopecia	Cameroon, Central Africa	Dubuis et al. (2003)
Tufted capuchin (*Cebus apella*)	*C. albicans*	Progressive weight loss, ulcerative and desquamative skin lesions	Rio Grande do Sul, Brazil	Cleff et al. (2008)
Venezuelan red howler monkey (*Alouatta seniculus*); Night monkey (*Aotus* sp); White-fronted capuchin (*Cebus albifrons*)	*C. albicans*	Alopecia lesions, erythematous and scaly plaques, or areas of skin that showed positive fluorescence under a Wood's lamp	Valledupar, Colombia	Echeverri De La Hoz et al. (2020)
Rodents and lagomorphs				
Red squirrel (*Sciurus vulgaris*)	*C. albicans*	Poor body condition score; mucous membranes at the base of the tongue and larynx were brownish, raised, and irregular. Esophagus thickened and inflamed, mucosa pale and necrotic. Liver pale, spleen pale and reduced. The body was infested with sucking lice, species *Neohaematopinus sciuri.* hemolytic *E. coli* and both α-hemolytic and non-haemolytic *Streptococcus* species were also isolated	Scotland	Simpson et al. (2009)
Brazilian porcupine (*Coendou prehensilis*)	*C. albicans*	Generalized infection. Microabscesses with bacteria – Enterobacteria and *Streptococcus hyointestinalis—*were present in the lungs, liver, spleen, kidneys, and lymph nodes, from which the yeast was isolated	Fortaleza, Ceará, Brazil	Castelo-Branco et al. (2013)

**Fig. 2:**
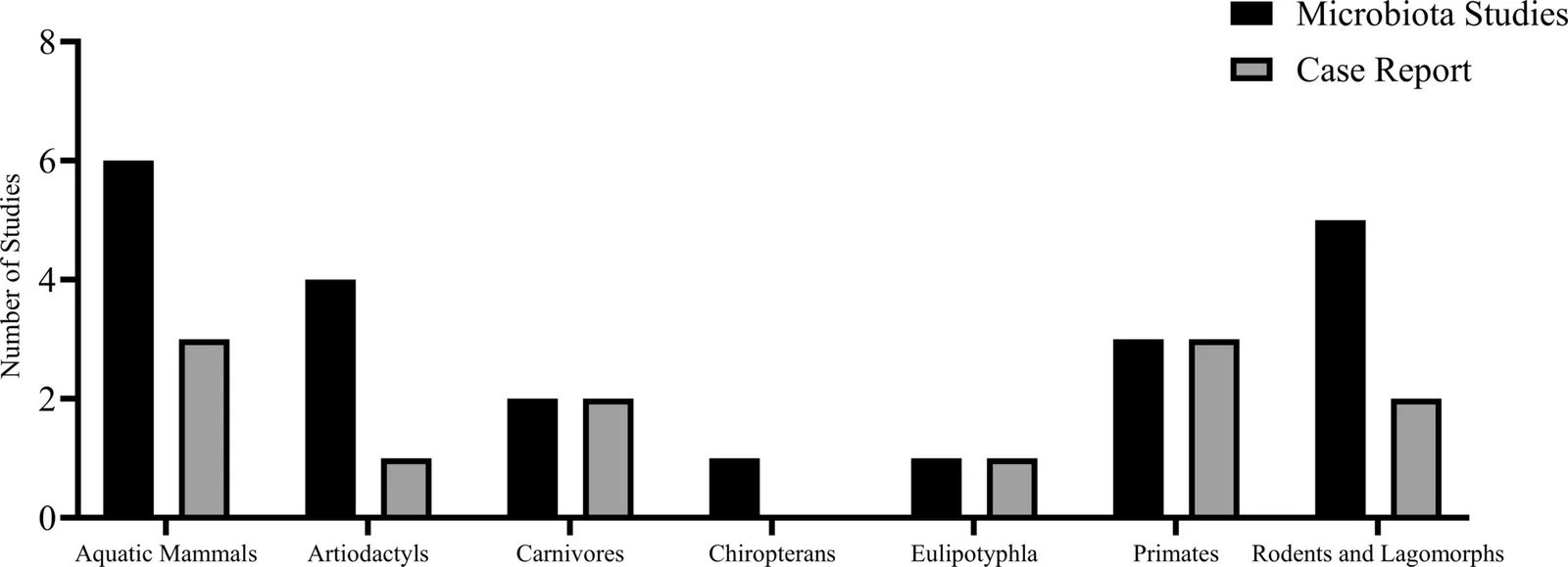
Distribution of the identified studies by wild animal groups

A total of 34 studies focused on mammal species, which were categorized as aquatic mammals (*n* = 9; 26.47%), artiodactyls (*n* = 5; 14.70%), carnivores (*n* = 4; 11.76%), chiropterans (*n* = 1; 2.94%), eulipotyphla (*n* = 2; 5.88%), primates (*n* = 6; 17.64%), rodents and lagomorphs (*n* = 7; 20.58%) (Fig. 2). Altogether, these studies recorded 74 distinct mammal species.

**Fig. 3:**
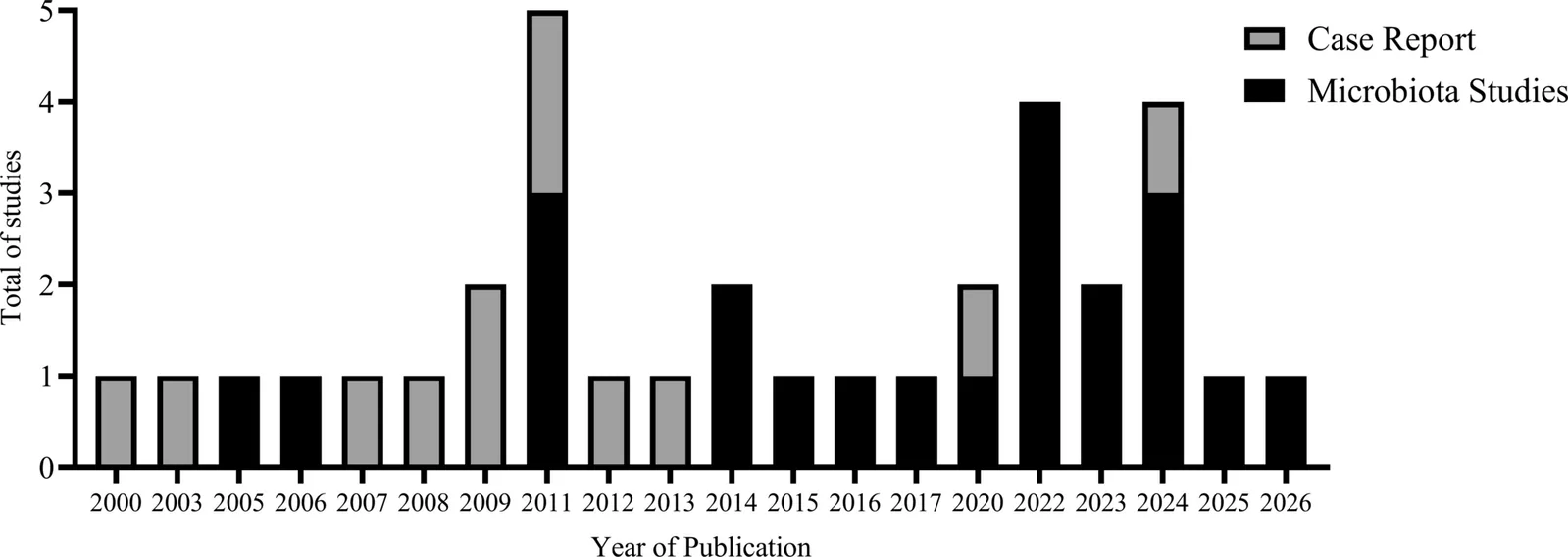
Compiled studies and their respective years of publication

The temporal distribution of the included studies (*n* = 34) spanned from 2000 to 2026 (Fig. 3). A higher concentration of publications was observed in 2011 (*n* = 5; 14.70%), 2022 (*n* = 4; 11.76%), and 2024 (*n* = 4; 11.76%), which together account for 38.22% of the total evidence regarding *Candida* spp. in wild mammals.

**Fig.4:**
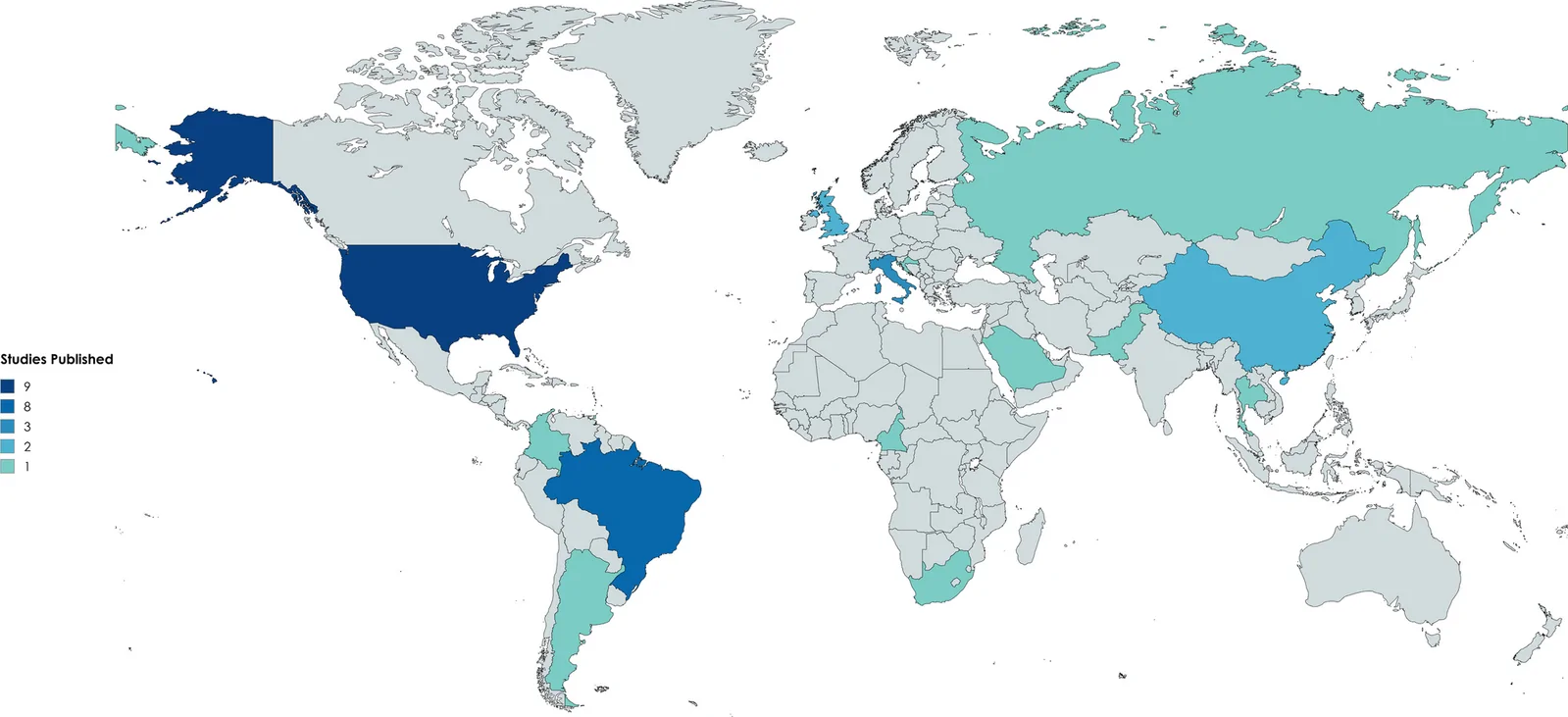
Distribution map of the selected studies included in this review

Regarding geographic distribution (Fig. 4), the studies were distributed across five continents, demonstrating a global interest in *Candida* spp. in wildlife. The Americas accounted for the majority of the records (*n* = 20; 58.82%), led by the United States (*n* = 9; 26.47%) and Brazil (*n* = 8; 23.53%), with additional reports from Argentina, Colombia, and Trinidad and Tobago (*n* = 1; 2.94%, each). Europe followed with 7 studies (20.59%), primarily from Italy (*n* = 3; 8.82%), the United Kingdom (*n* = 2; 5.88%), Croatia (*n* = 1; 2.94%), and Russia (*n* = 1; 2.94%). Research in Asia and the Middle East comprised 5 studies (14.71%), including contributions from China (*n* = 2; 5.88%), Saudi Arabia, Pakistan, and Thailand (*n* = 1; 2.94%, each). Finally, the African continent was represented by 2 studies (5.88%) from South Africa and Cameroon.

Regarding *Candida*, 21 species were identified across the selected studies (*n* = 34), although in some publications (*n* = 7; 20.58%), identification was not reported at the species level. The laboratory strategies for *Candida* isolation and identification were assessed across all included studies. Microbiological culture was the most frequent primary isolation method, reported in 28 studies (82.35%). Phenotypic and biochemical characterization, which integrated traditional biochemical assays, phenotypic tests, commercial API® kits, and automated Vitek® 2 systems, was utilized in 14 (41.17%) publications. Molecular techniques were identified as PCR (*n* = 10; 29.41%) and genetic sequencing (*n* = 9; 26.47%). Proteomic identification via MALDI-TOF MS was reported in 4 (11.76%) studies. Complementary diagnostic approaches, such as histopathology and cytology, were performed in 8 (23.52%) works, while 2 (5.88%) studies provided no detailed information regarding the laboratory protocols.^1^

In studies focusing exclusively on microbiota (*n* = 22), the most frequent sampling sites were the gastrointestinal tract, including anal/rectal swabs, fecal samples, guano or bolus (*n* = 14; 63.64%) and the oral cavity (*n* = 9; 40.91%). Respiratory surfaces were assessed through nasal mucosa (*n* = 7; 31.82%) and blowhole swabs (*n* = 7; 31.82%). Samples from integumentary system (skin, hair and quills) were reported in 3 studies (13.65%). Other sites included the genital tract (*n* = 2; 9.09%), lung tissues (*n* = 1, 4.55%), ocular surfaces (*n* = 1; 4.55%), and gastric fluid (*n* = 1; 4.55%).

**Fig. 5:**
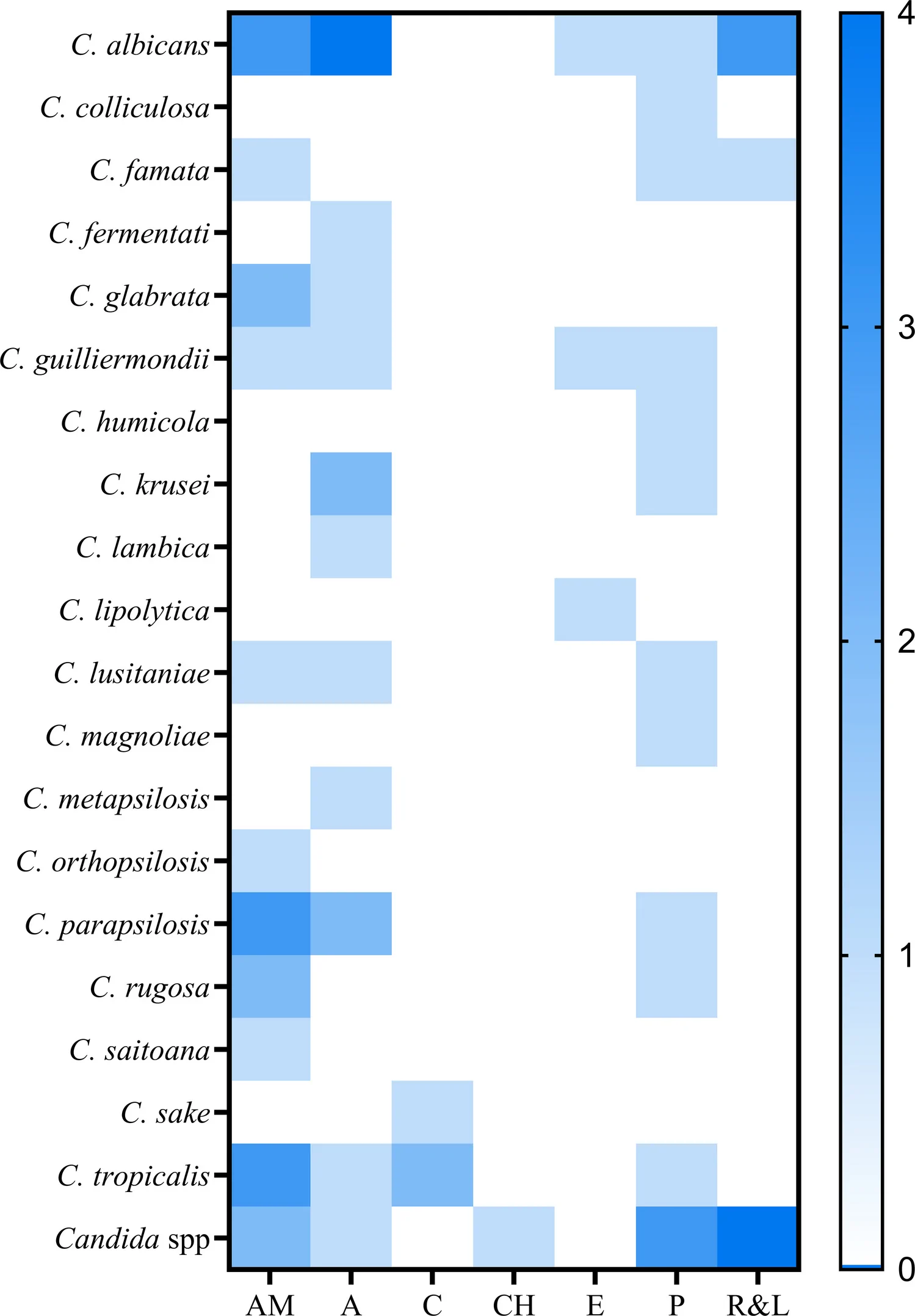
Frequency of *Candida* spp. identified in the microbiota of wild mammals from the selected studies. Aquatic mammals (AM); artiodactyls (A); carnivores (C); chiropterans (CH); eulipotyphla (E); rodents and lagomorphs (R&L); primates (P)

*C. albicans* was the most prevalent species in microbiota reports, identified in 12 investigations (54.55%). Other frequently isolated taxa included *C. tropicalis* (*n* = 7; 31.82%), *C. parapsilosis* (*Lodderomyces parapsilosis*) (*n* = 6; 27.27%), and *C. guilliermondii* (*Meyerozyma guilliermondii*) (*n* = 4; 18.18%). Species such as *C. krusei* (*Pichia kudriavzevii*), *C. glabrata* (*Nakaseomyces glabratus*), *C. famata* (*Debaryomyces hansenii*), *C. rugosa* (*Diutina rugosa*), and *C. lusitaniae* (*Clavispora lusitaniae*) were each documented in 3 studies (13.64%). Several species were reported in only one study (4.55% each), including *C. lipolytica* (*Yarrowia lipolytica*), *C. orthopsilosis*, *C. humicola* (*Vanrija humicola*), *C. colliculosa* (*Torulaspora delbrueckii*), *C. magnoliae* (*Entelexis paramagnoliae*), *C. saitoana* (*Suzukiozyma candida*), *C. fermentati* (*Meyerozyma caribbica*), *C. lambica* (*Pichia fermentans*), *C. metapsilosis*, and *C. sake* (*Fermentozyma sake*) and *C. slooffiae* (*Kazachstania slooffiae*) (Liu et al. 2026) (Fig. 5).

Regarding the species identification, 50% of the microbiota investigations (*n* = 11; 50.00%) reported isolates simply as *Candida* spp. However, a distinction was noted in the reporting patterns: in five studies (22.73%), *Candida* spp. was the only taxonomic designation provided, with no species-level identification achieved. In contrast, the remaining six studies (27.27%) reported unidentified *Candida* isolates alongside other successfully identified species.

**Fig. 6:**
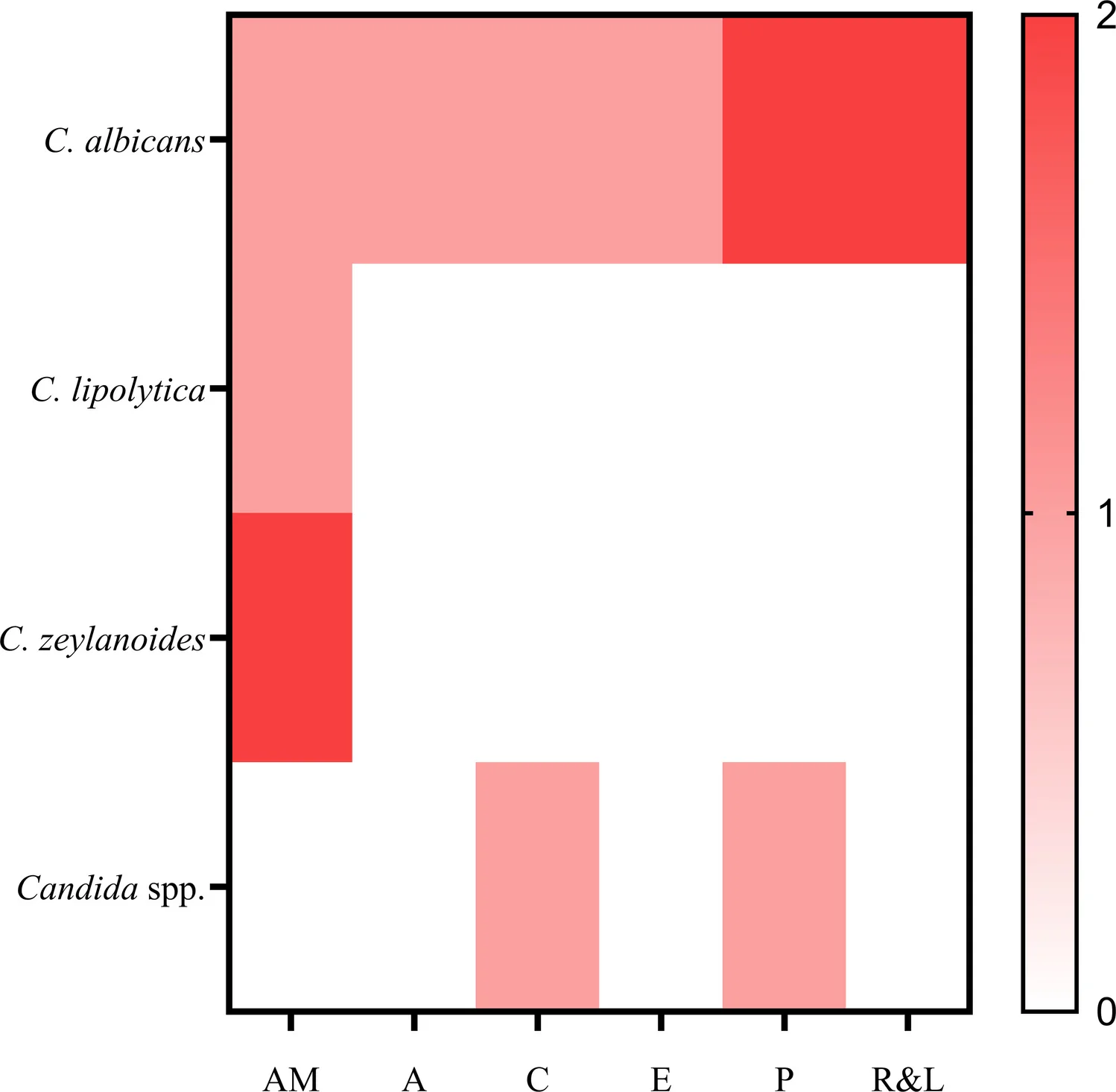
Frequency of *Candida* spp. identified in infectious processes from the selected studies. Aquatic mammals (AM); artiodactyls (A); carnivores (C); eulipotyphla (E); rodents and lagomorphs (R&L)

As for studies related to infectious processes (*n* = 12), *Candida albicans* was the most prevalent species, identified in 8 out of 12 investigations (66.67%). This was followed by *C. zeylanoides* (*Dujonia zeylanoides*) (*n* = 2; 16.67%) and *C. lipolytica* (*n* = 1; 8.33%), while two studies (16.67%) did not specify the *Candida* species involved (Fig. 6).

The clinical manifestations associated with *Candida* infection were diverse and often severe. Oral and upper digestive tract lesions were prominent, including necrotic glossitis, stomatitis, ulcerative pharyngitis, and esophageal inflammation. Cutaneous involvement was also frequent, characterized by fungal dermatitis, ulcerative or desquamative lesions, erythematous plaques, and alopecia. Other localized manifestations included mycotic rhinitis, ulcerative keratitis, and necrotizing pyelonephritis. Systemic or generalized infections with the presence of microabscesses in the lungs, liver, spleen, and kidneys were documented in several cases, often leading to death.

Notably, only five reports (41.67%) described the exclusive isolation of *Candida* spp. In the majority of cases (*n* = 7; 58.33%), the authors reported the presence of other pathogens or underlying conditions. These included other fungi (e.g., *Aspergillus* spp., *Filobasidiella neoformans var. neoformans* (*Cryptococcus neoformans*)*, Malassezia* sp., *Pneumocystis* sp., and *Trichophyton mentagrophytes*) and bacteria (e.g., *Staphylococcus* sp., *Escherichia coli*, *Clostridium* sp., and *Campylobacter coli*). Furthermore, viral co-infections such as Canine Distemper Virus (CDV) and Porcine Circovirus type 2 were identified, as well as an infestation by the sucking louse *Neohaematopinus sciuri*.

Antifungal susceptibility testing was performed in 5 of the total studies (14.70%), evaluating drugs from the polyene, azole, echinocandin, and pyrimidine analogue classes. The susceptibility profiles varied significantly across the sample. While 100% susceptibility to all tested drugs (including polyenes, azoles, and echinocandins) was reported in one investigation, in four studies were identified some degree of reduced susceptibility or resistance. Resistance to azole derivatives, specifically fluconazole and itraconazole, was the most frequent finding, with isolates in some cases being non-susceptible to up to four different agents within this class. Although sensitivity to amphotericin B and echinocandins (caspofungin, micafungin, and anidulafungin) was generally preserved in most reports, one study documented a broad profile of low susceptibility encompassing all tested antifungal classes.

Environmental and anthropogenic factors associated with the presence of *Candida* spp. were discussed in 11 of 34 studies (32.35%). These factors were categorized into: (1) Anthropogenic pressure and residues, including contact with human garbage, urban pollution, and contamination by human waste; (2) Agricultural and industrial impact, highlighting exposure to azoles used in farming, oil and gas exploration, and industrial runoff; (3) Captivity and management conditions, where diet, antibiotic use, and environmental stress were associated with a loss of microbial diversity and shifts in the commensal mycobiota. One study (2.94%) reported that robust conclusions regarding the influence of biological and ecological variables could not be drawn from the available correlations and statistical analyses, owing to the small sample size, and emphasized the need for further research.

## Discussion

The present review reveals the ubiquitous and diverse presence of yeasts from the genus *Candida* in 74 wild mammal species, spanning from polar to tropical environments. Although it is widely known that these yeasts are found in animals, veterinary medicine still appears to underestimate fungi in general, and the genus discussed here in particular, as potential emerging pathogenic agents. They are frequently neglected in favor of other microbes, such as bacteria and viruses. These data demonstrate a scarcity of studies involving wildlife, as well as the ecological role of these fungi, acting both as constituents of the commensal microbiota and as pathological agents in various infectious processes.

It is worth noting that most research involving *Candida* in wild animals has focused on avian species (Reis et al. 2019; Silva et al. 2019; Talazadeh et al. 2022). This trend may be related to the ease of sample collection, the higher thermotolerance observed in some avian species, and the relevance of classic fungal diseases such as cryptococcosis in urban pigeons (Sirag et al. 2021). However, not all *Candida* species are thermotolerant, which limits their pathogenicity in homeothermic hosts.

The increase in publications, particularly between 2022 and 2024, coincides with rising global awareness of fungal pathogens, driven by the WHO Fungal Priority Pathogens List (2022). Concurrently, the SARS-CoV-2 pandemic highlighted the importance of monitoring wildlife populations and their constituent microbial communities. Consequently, there has been a notable advancement in fungal identification techniques using MALDI-TOF and sequencing, alongside a growing interest in the field of mycology.

The concentration of research in the United States of America (*n* = 9; 26.47%) and Brazil (*n* = 8; 23.53%) reflects a combination of advanced research infrastructure and high levels of mammalian biodiversity, respectively. However, despite the expanded sample size of this review (*n* = 34), a significant geographic imbalance remains. Regions in Asia (*n* = 5; 14.71%) and Africa (*n* = 2; 5.88%) are still underrepresented in the global ecological landscape of the genus *Candida*. Given that these continents are home to some of the world's most biodiverse ecosystems, which remain significantly understudied regarding wildlife mycology, they likely represent 'hotspots' for both undiscovered *Candida* species and emerging resistant lineages. The overall diversity of mammal hosts and identified fungal taxa across five continents highlights the remarkable ecological plasticity of these yeasts.

The variability, characterized by the identification of 21 distinct species, is attributed to the ability of these yeasts to colonize different niches within the organism, supported by significant genomic plasticity and a highly adaptable metabolic profile. Such features allow for rapid physiological adjustment to diverse microenvironments and nutrient availability, which ultimately facilitate adherence and tissue invasion (Mba and Nweze 2020).

However, under conditions of host immunosuppression or environmental stress, these yeasts may become pathogenic (Alves et al. 2017). Moreover, it is likely that the true fungal diversity remains underestimated; nearly 50% of the microbiota investigations reached only genus-level identification (*Candida* spp.), often due to the limitations of traditional phenotypic methods in distinguishing cryptic species (Fontecha et al. 2019). This taxonomic gap could suggest that the 21 species identified may represent only a fraction of the actual mycobiota from these animals. We also suggest that this lack of definition may mask the presence of new or emerging species, which remain hidden under a generic designation.

Infections caused by the genus *Candida* are predominantly secondary, arising when the host's equilibrium is disrupted. As observed in selected studies, co-isolation with other pathogens such as Porcine Circovirus (Zlotowski et al. 2011) and *Trichophyton mentagrophytes* (Pollock and Ramsay 2000) is consistent with the role of factors such as environmental stress, inadequate nutrition, and antimicrobial therapies in triggering significant dysbiosis. These alterations in the natural microbiota reduce bacterial competition and favor colonization and tissue invasion by yeasts. In this context, the presence of *Candid*a often acts as a marker of immunological vulnerability, where the fungus exploits the host's weakened state to exacerbate the clinical picture. However, its opportunistic nature does not exclude its potential as a primary pathogen in scenarios of high vulnerability. A clear example of this aggressiveness was observed in the report by Cleff et al. (2008), in which *C. albicans* was isolated as a monoinfectious in skin lesions and in various internal organs. Isolation of the fungus from sterile sites and in a disseminated pattern, in the absence of other infectious agents, suggests that the virulence of *C. albicans*, combined with intrinsic host factors, is sufficient to overcome immune barriers and cause fatal systemic candidiasis.

*Candida albicans* remains the most frequently identified species in both microbiota and pathological process studies. This finding may reflect the global interest in this species, as well as the specificity and ease of the laboratory tests used for its identification. Nevertheless, other species such as *C. krusei* and *C. tropicalis* have already been described in infections of hospitalized human patients (Cheong et al. 2025) as well as *C. glabrata* and *C. parapsilosis* in dogs and cats (Lamm et al. 2013; Woo et al. 2017; Geum et al. 2024). Interestingly, *C. zeylanoides* was the second most frequently isolated species after *C. albicans* in the published clinical reports, specifically in aquatic animal species. A targeted search in the databases revealed that related publications about this species primarily involve products of animal origin (Spampinato et al. 2022; Geronikou et al. 2023; Liu et al. 2025) and biotechnological applications (Mitrea et al. 2019; Sayın Börekçi et al. 2022).

A particularly concerning finding is the emergence of *Candida auris* outside the nosocomial environment. Although initially recognized as a multidrug-resistant human pathogen associated with high mortality in hospital settings (Dang-Vu et al. 2024), recent evidence highlights its presence in diverse wildlife hosts. Notable reports include the isolation of this species from dolphins (Ferrara et al. 2025) and from snakes (Cafarchia et al. 2024) suggesting a broader environmental distribution than previously understood. The detection of this multidrug-resistant pathogen in wild populations represents a critical One Health milestone, as it implies that wildlife may act as sentinels or potential reservoirs for highly resistant lineages, potentially driven by anthropogenic environmental shifts and global warming (Casadevall et al. 2019).

The heterogeneity in antifungal susceptibility profiles observed in this review suggests varying levels of environmental or anthropogenic pressure on wild mammalian populations. The prevalence of azole resistance is a particularly concerning finding, as these compounds are widely used not only in human and veterinary medicine but also as fungicides in agricultural practices (Williams et al. 2024). In our search, the number of studies that analyzed the susceptibility profile in this animal population was very small, indicating a scarcity of data, and therefore the hypotheses raised suggest that the resistance observed in wildlife isolates may be a bioindicator of environmental contamination. The contrast between studies showing full susceptibility (Brustenga et al. 2025) and those reporting multi-resistance highlights a potential gradient of exposure: animals in more preserved habitats may harbor sensitive strains, while those in human-altered environments or rehabilitation centers may act as reservoirs for resistant strains (Castelo-Branco et al. 2013; Rhimi et al. 2022).

Climate change and anthropogenic actions such as deforestation, habitat fragmentation, pesticide use, and increasing urbanization directly impact the composition and stability of animal microbiota. These environmental changes create conditions of ecological imbalance that may favor the growth of opportunistic microorganisms or induce the selection of more resistant strains. In fungi, this phenomenon has been documented in several situations, suggesting that yeasts can respond rapidly to changing environmental conditions (Case et al. 2022; Seidel et al. 2024).

In this context, yeasts of the genus *Candida* may also serve as indicators of environmental quality (Brilhante et al. 2015; Rana et al. 2023). Alterations in the antifungal susceptibility profile of some strains may reflect the presence of chemical residues in the environment, such as pesticides, heavy metals, and agricultural antifungals. Among the studies included in this review, that of Rhimi et al. (2022) stands out: the authors evaluated strains obtained from wild boars and observed an absence of resistance to the main antifungals tested. They suggest that this susceptibility indicates an environment free of significant chemical contamination, positioning these yeasts as potential markers of preserved environments. This approach, though still underexplored, contributes to strengthening environmental mycology as a tool for ecological surveillance, allowing for the inference of selective pressures even in the absence of clinical alterations in the hosts.

Conducting this systematic review revealed significant challenges inherent to the study of *Candida* in wildlife. A primary obstacle is the overall scarcity of research focusing specifically on wild mammal populations, which is often compounded by a lack of standardized identification protocols and geographical disparities in data collection. Nevertheless, these limitations reinforce the importance of expanding mycological studies through an integrative approach that incorporates clinical, molecular, and ecological data. Active surveillance of the fungal microbiota in wild animals should be considered a fundamental component of environmental conservation, biosecurity, and One Health strategies. Furthermore, characterizing strains regarding antifungal resistance and virulence factors is essential for mapping areas under greater anthropogenic influence and anticipating potential threats to both public and animal health.

## Conclusion

The present systematic review reveals that the genus *Candida* is a ubiquitous and highly adaptable component of the wild mammal mycobiota, with 21 distinct species identified across diverse ecological niches. While *C. albicans* remains the predominant taxon, the identification of a wide array of non-albicans species highlights remarkable ecological plasticity. However, the high frequency of unidentified isolates (50%) and the significant geographic imbalances, particularly the lack of data from Africa and Asia, demonstrate that our current understanding of wildlife mycology is still fragmented. To transition from opportunistic reporting to a structured global surveillance model, it is imperative to move beyond traditional phenotypic methods and adopt MALDI-TOF MS or ITS region sequencing as the minimum identification standard. Furthermore, future research must prioritize the integration of antifungal susceptibility testing (AST) using Minimum Inhibitory Concentration (MIC) assays with standardized international breakpoints (CLSI or EUCAST) to ensure data comparability. Methodological harmonization is equally essential, including the use of amplicon sequencing to characterize fungal communities that escape traditional culture-dependent techniques. By addressing these gaps and integrating wildlife data into One Health surveillance frameworks, the scientific community can better monitor how anthropogenic pressures influence fungal diversity and effectively map the ecological dynamics of the genus *Candida* in natural environments.

## Supplementary Information

Below is the link to the electronic supplementary material.

Supplementary file1 (XLSX 8 KB)

## Data Availability

No datasets were generated or analysed during the current study.

## Footnotes

1 The cumulative percentage exceeds 100% due to the inclusion of studies that utilized multiple identification methods (e.g., culture combined with molecular and phenotypic tests). The denominators are specific for each analysis, such as 22 for microbiota and 12 for infection reports, as example.
